# MAS-UNet: a U-shaped network for prostate segmentation

**DOI:** 10.3389/fmed.2023.1190659

**Published:** 2023-05-18

**Authors:** YuQi Hong, Zhao Qiu, Huajing Chen, Bing Zhu, Haodong Lei

**Affiliations:** ^1^School of Computer Science and Technology, Hainan University, Haikou, China; ^2^Hainan Provincial Public Security Department, Haikou, China; ^3^Haikou Hospital of the Maternal and Child Health, Haikou, China

**Keywords:** UNet, attention gate, ASPP, prostate, channel attention, spatial attention

## Abstract

Prostate cancer is a common disease that seriously endangers the health of middle-aged and elderly men. MRI images are the gold standard for assessing the health status of the prostate region. Segmentation of the prostate region is of great significance for the diagnosis of prostate cancer. In the past, some methods have been used to segment the prostate region, but segmentation accuracy still has room for improvement. This study has proposed a new image segmentation model based on Attention UNet. The model improves Attention UNet by using GN instead of BN, adding dropout to prevent overfitting, introducing the ASPP module, adding channel attention to the attention gate module, and using different channels to output segmentation results of different prostate regions. Finally, we conducted comparative experiments using five existing UNet-based models, and used the dice coefficient as the metric to evaluate the segmentation result. The proposed model achieves dice scores of 0.807 and 0.907 in the transition region and the peripheral region, respectively. The experimental results show that the proposed model is better than other UNet-based models.

## 1. Introduction

According to statistics from the National Cancer Institute, in 2017, there were 161,360 new cases of cancer and 26,730 deaths that were related to cancer in America, indicating that prostate cancer has always been a major threat to men's health. Effective segmentation of the prostate and its different regions is helpful to predict the pathological stage and check the therapeutic effect (1). Compared with CT, magnetic resonance imaging (MR) does no harm to the human body and it also has great tissue contrast and better resolution (2). On account of these advantages, it has become the mainstream imaging method for prostate region evaluation (3).

The segmentation of the prostate region in MR images is ordinarily performed by radiologists based on visual examination of the image slices. Manual segmentation requires superb technology and full concentration, and it is time-consuming and prone to deviations within and between operators, which is not suitable for the segmentation of a large number of samples. Therefore, there is an urgent need for reliable automatic segmentation methods for prostate MRI images. However, segmentation of the prostate region is quite challenging because the size and shape of glands in prostate MRI images often have large variability. In addition, the heterogeneity of the signal intensity around the rectal coil, the low contrast between the gland and the adjacent structure, and the anisotropic spatial resolution are reasons for the difficulty of prostate segmentation (4, 5).

The automatic segmentation of prostate regions is an earlier research topic. In recent years, with the improvement of hardware performance and the continuous development of deep learning-related technologies, the method based on convolutional neural networks (CNNs) has gradually replaced the traditional method. Because the deep learning method can learn complex features and accurately classify pixels, the segmentation results are obtained (6), and the segmentation result is generally better than the traditional method. Some studies have proposed several deep learning-based methods for prostate segmentation, such as the classical U-Net (7) model, which is the basis of many recent literature and research, as well as the MultiResU-Net (8), density-UNet (9), and Attention UNet (10) models. Though these models have achieved decent results in prostate segmentation, there is still possibility for further improvement.

In view of the above problems, we proposed a U-shaped structure network for prostate region segmentation. Our main contributions in this study are as follows:

1. Based on Attention U-Net, this study proposes to add channel attention to the network to further clarify the importance between channels, so that the network ignores secondary information and focuses more on important channels to extract features better.
2. This study introduces an ASPP structure at the end of the encoder in the U-shaped structure network.
3. In order to reduce the hardware requirements of model training and make the model achieve better performance than previous models while the batchsize is small, this study uses GN to replace the commonly used BN.
4. In order to prevent overfitting, this study introduces dropout in the last downsampling process of the encoder part of the network. Experiments show that it can effectively improve overfitting and further improve segmentation performance.
5. In this study, through the comparison experiments with Unet, Attention U-Net, UNet++, R2Attention U-Net, and Res-UNet, it is proved that the model proposed in this study has better performance than the traditional models mentioned above in prostate segmentation.

## 2. Related study

FCN (11) is a pioneer of image segmentation, which makes full use of convolution to extract features from images. On the basis of FCN, a classical encoder–decoder model U-Net (7) is proposed for medical image segmentation tasks, which achieved decent results on various segmentation tasks.

Most models for medical image segmentation are improved based on U-Net, such as UNet++ (12), Attention-UNet (10), Res-UNet (13), Dense-UNet (14), SA-Net (15), Bio-Net (16), and MRF-UNet (17). UNet++ replaces the clipping and splicing operations of the U-Net direct connection part with convolution operations, obtaining better feature information and making up for the information loss caused by sampling. Attention-UNet uses attention gates to give more importance to the key region of the feature map and make the network more focused on goals. In order to further reduce the loss of information and improve performance, Res-UNet and Dense-unet use Res-block in ResNet (18) and density-block in DenseNet (19) instead of ordinary convolution. SA-Net is a lightweight network, in which a spatial attention module is applied at the end of the encoder. Bio-Net adds backward skip connections to the network so that the feature information from the decoder can be transmitted back to the encoder and aggregated with the feature information in the encoder. MRF-UNet combined UNet with Markov random field, which achieved better performance on out-of-distribution data than the original UNet.

The attention mechanism is usually used for natural image analysis, knowledge graphs, natural language processing, automatic image annotation (20), machine translation (21), and classification tasks (22). The trainable attention mechanism is divided into hard attention and soft attention. The hard attention mechanism is usually non-differentiable and relies on reinforcement learning to update parameters, which makes the training process of the model more difficult. According to Ypsilantis and Montana (23), recursive hard attention was used to detect abnormalities in chest X-ray scans. On the contrary, the soft attention mechanism can be trained using standard backpropagation. For example, additive soft attention is used for sentence translation (24) and image classification (22). According to Hu et al. (25), channel attention was used to highlight important feature dimensions, which achieved the best performance in the ILSVRC 2017 image classification challenge. In addition, some people have proposed self-attention technology to eliminate the dependence on external sector control information. For example, Wang et al. (26) used a non-local self-attention mechanism to capture deep dependencies. According to Jetley et al. (22), self-attention is used to perform class-specific pooling to obtain more accurate and robust image classification performance.

In traditional DCNN, there are a series of problems in upsampling and downsampling. On the one hand, the internal data structure and spatial hierarchical information are lost due to pooling. On the other hand, the data of small objects (under certain conditions) will be lost after downsampling, meaning the information cannot be reconstructed. This problem is particularly significant in semantic segmentation, and dilated convolution is proposed to solve these problems. Dilated convolution can arbitrarily expand the receptive field without introducing additional parameters, and a larger receptive field can improve the effect of small object recognition and segmentation in the task of target detection and semantic segmentation. ASPP (27) module uses multiple parallel atrous convolutions (dilated convolution) layers with different dilation rates, which do achieve decent results in many segmentation tasks.

## 3. Preliminaries

### 3.1. UNET

The UNet network consists of an encoder and a decoder. The encoder part follows the classical structure of convolution networks. The convolution block consists of two repeated 3 × 3 convolutions. Each convolution is followed by a ReLU activation function and a 2 × 2 maximum pooling operation with a step of 2 for downsampling. In each downsampling step, the number of feature channels is doubled. Each step in the decoder upsamples the size of feature maps by 2, and the number of feature map channels is reduced to half using 2 × 2 convolution (deconvolution). Feature maps from the encoder are directly passed to the decoder with skip connections. After concatenation, there is a convolution block to reduce the number of channels. At the end of the decoder, there is a 1 × 1 convolution layer which is designed for the output.

UNet obtains its energy function by combining the pixel-level softmax function calculated for the last layer of the feature map with the cross-entropy loss function. The definition of the softmax function is as follows:


(1) $$p_{k}(x)=\exp(a_{k}(x))/(\Sigma_{k^{\prime }=1}^{K}\exp(a_{k^{\prime }}(x))$$

where represents the activation function in feature channel k at the pixel position *x* ∈ Ω, Ω ⊂ ℤ^2^. *K* is the number of classes and *p*_*k*_(*x*) is the approximated maximum-function. *p*_*k*_(*x*) ≈ 1 for the *k* that has the maximum activation *a*_*k*_(*x*), and *p*_*k*_(*x*) ≈ 0 for all other *k*. The cross entropy then penalizes at each position the deviation of *p*_*l*(*x*)_(*x*) from 1 using:


(2) $$\begin{array}{l} E=\underset{x\in \Omega }{\Sigma }w\left(x\right)\log\left(p_{l\left(x\right)}\left(x\right)\right) \end{array}$$

where *l* : Ω → {1, ..., *K*} is the true label of each pixel, and *w* : Ω → ℝ is a weight map which can make some pixels more important than the others while training (7).

### 3.2. Attention gate

Attention gate is a mechanism that can be merged into any existing CNN architecture. Let $x^{l}=\left\{x_{i}^{l}\right\}$ be the activation map of the chosen layer *l* ∈ {1, ..., *L*}, where each $x_{i}^{l}$ represents a pixel-by-pixel feature vector of length *F*_*l*_ (i.e., the number of feature-maps in layer l). For every $x_{i}^{l}$, AG will calculate the coefficient $\alpha^{l}={\left\{\alpha_{i}^{l}\right\}}_{=1}^{n}$, $\alpha_{i}^{l}\in \left[0,1\right]$, in order to identify the key region of the feature map and only reserve the parts that are related to specific tasks. The output of the attention gate is:


(3) $$\begin{array}{l} x^{l}={\left\{\alpha_{i}^{l}x_{i}^{l}\right\}}_{i=1}^{n} \end{array}$$

in which each vector is scaled by the corresponding attention coefficient (10).

## 4. Materials

The prostate dataset used in this study is the Task-05 prostate data set of the MSD competition, including 48 sets of multimodal MRI data, provided by Radboud University (Netherlands). Each set of data includes two modalities: transverse t2-weighted scan (resolution 0.6 × 0.6 × 4 mm) and apparent diffusion coefficient (ADC) map (2 × 2 × 4 mm). A total of 80% of the data have manual segmentation labels, including two prostate regions: transition zone (TZ) and peripheral zone (PZ).

## 5. Methods

### 5.1. Architecture

**Figure 1:**
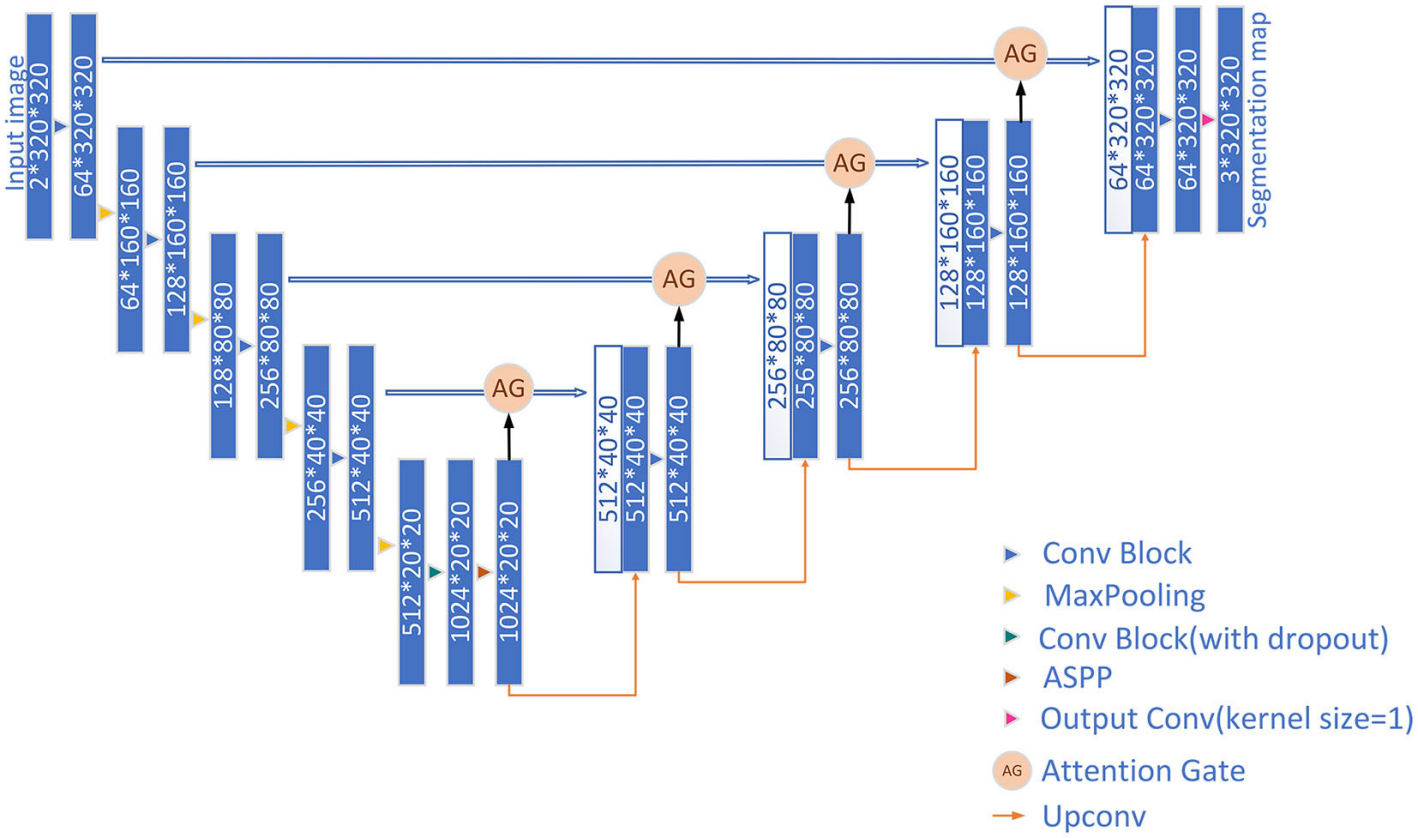
Structure of the proposed network.

In this study, inspired by the UNet framework, a new prostate segmentation network is proposed. The architecture of the whole network is shown in Figure 1. First, the first half of the network (i.e., encoder) is used to extract features from 2d slices of MRI images of 3d tissues. The second half (i.e., decoder) is then used to generate the predicted segmentation results, where each type of label is segmented in different channels.

The network proposed in this article consists of two parts, an encoder and a decoder. The encoder consists of five convolution blocks, four Max Pooling blocks, and a spatial dilated convolution pyramid (ASPP) module. In the first four convolution blocks, each convolution block consists of two 2d convolution layers followed by group normalization (GN) and ReLu activation function. The fifth convolution block adds a dropout layer on the basis of the first four to prevent overfitting. An ASPP module is added at the bottom of the encoder to further extract features. Each Max Pooling block performs a maximum pooling to achieve downsampling of the feature map by 2. The decoder consists of four upsampling modules, four attention gate (AG) modules, three convolution blocks, and a 2d convolution layer for output. Each upsampling module uses the nearest neighbor interpolation to upsample the feature map. The final 2d convolution layer is responsible for outputting segmentation results.

### 5.2. Convolution blocks

As for batch normalization (BN), it will get a decline in performance if the batchsize is too small; on the other hand, big batchsize will consume a lot of memory, especially when large-size images are input into the network. In order to get better segmentation results while the batchsize is small, we decided to use group normalization (GN) instead of BN.

**Figure 2:**
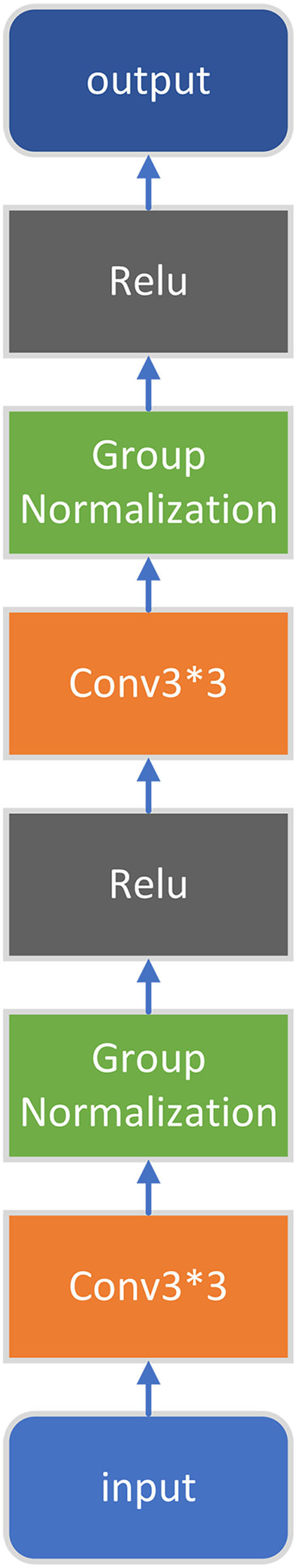
Conv block.

The structure of the first four convolution blocks and the convolution blocks used in the decoder part is shown in Figure 2.

Using group normalization solves the internal, covariate, and shift problems, and the result is much better than batch normalization when the batchsize is small (in our experiments the batchsize is set to 4). The definition of group normalization is:


(4) $$\begin{array}{l} y=\frac{x-E\left[x\right]}{\sqrt{Var\left[x\right]+\varepsilon }}*\gamma +\beta \end{array}$$

The input feature map x is divided into several groups according to the channel, and the mean and standard deviation of each group are calculated, respectively. γ and β are learnable parameters.

In the encoder part, the input image will go through four such convolution blocks to extract its features. After each of those four convolution blocks, the max pooling module will use the maximum pooling to downsample the feature map (by 2), the number of channels of the feature map remains unchanged, and the length and width become half of the original, so as to further extract features and reduce the number of parameters.

**Figure 3:**
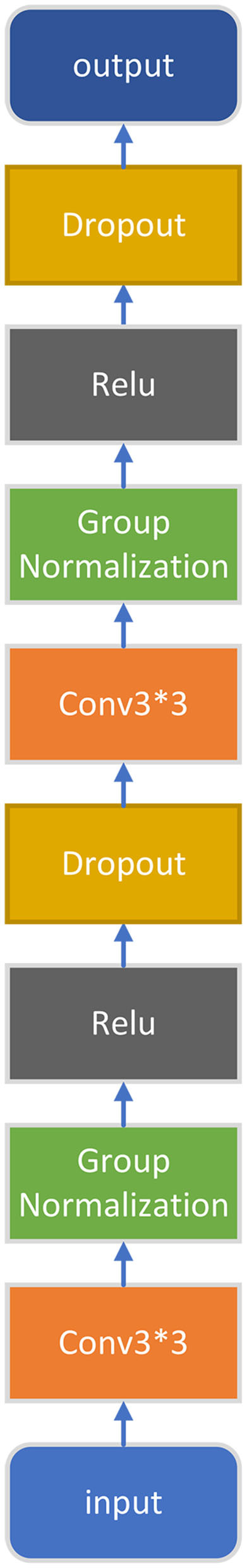
Conv block with dropout.

After four rounds of downsampling, the feature map comes to the fifth convolution block. The structure of the last convolution block in the encoder is shown in Figure 3. Compared with the first four convolution blocks, this convolution block has an additional Dropout layer (where the *p*-value is set to 0.5). Experiment results show that adding Dropout can effectively alleviate overfitting and improve the final segmentation results.

### 5.3. ASPP module

**Figure 4:**
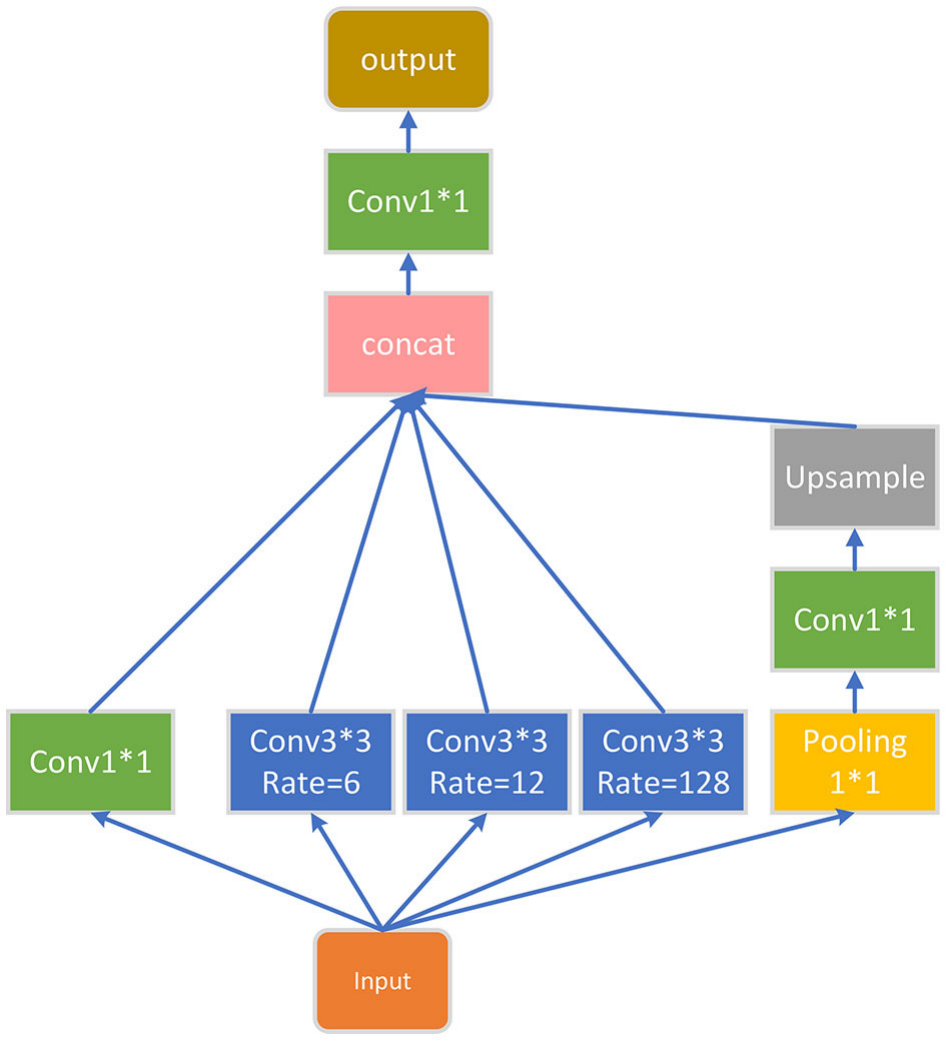
ASPP block.

The final part of the encoder is an ASPP module, which is added to extract further features, and its structure is shown in Figure 4. For the input feature map, ASPP uses dilated convolution with different dilation rates to process it (in this article, the dilation rates are set to 1, 6, 12, and 18), then concatenates the obtained results together, expands the number of channels, and finally reduces the number of channels to the desired value through a 1^*^1 convolution layer.

The algorithm of the ASPP module is as follows:

**Algorithm 1:**
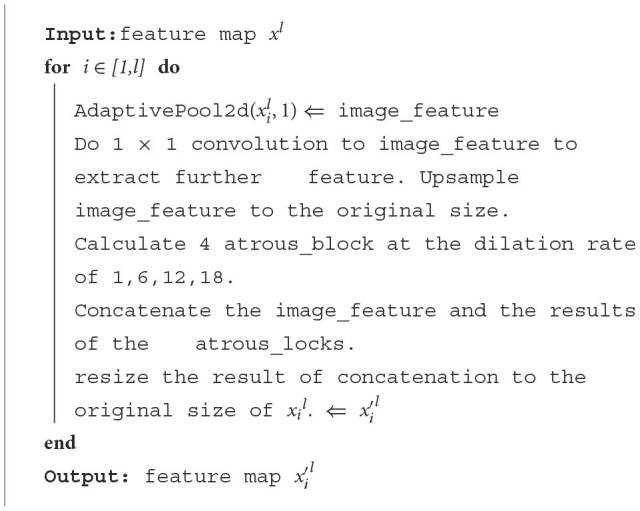
ASPP Block.

### 5.4. Upsampling

**Figure 5:**
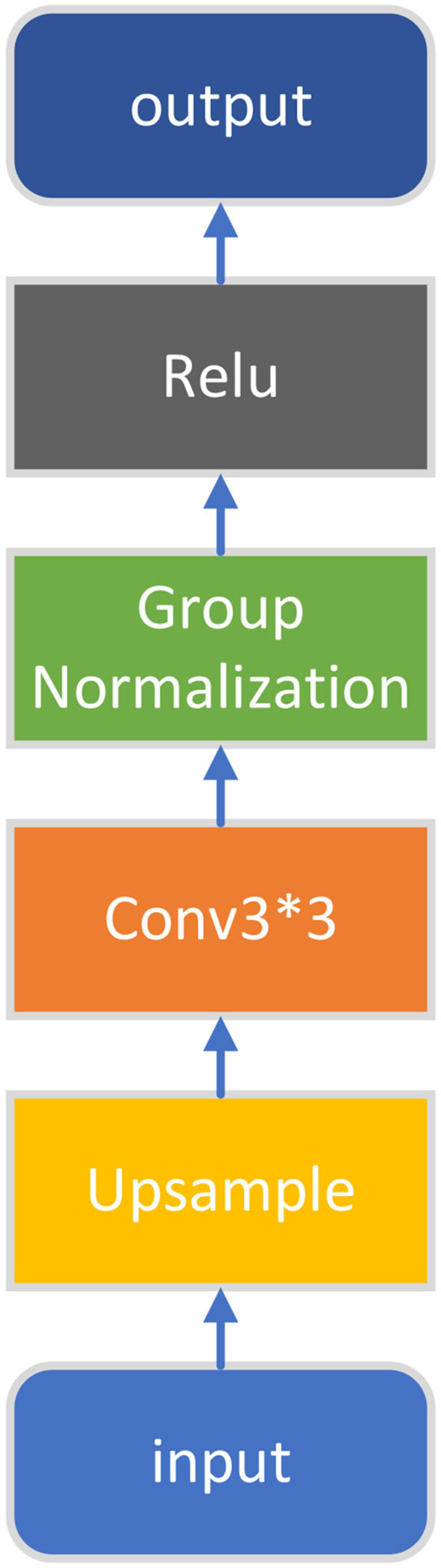
Up conv block.

The feature map obtained by the encoder will be sent to the decoder. The structure of the upsampling module is shown in Figure 5. We use the nearest neighbor interpolation for upsampling, which is defined as:


(5) $$\begin{array}{l} f\left(X,Y\right)=f\left(\frac{W}{w}*x,\frac{H}{h}*y\right) \end{array}$$

The size of the input feature map is W ^*^ H, and the size of the upsampled feature map is w ^*^ h. The pixel value of pixel (x, y) on the upsampled feature map equals the pixel value of pixel (W/w ^*^ x, H/h ^*^ y) on the original feature map.

### 5.5. Attention gate

In order to improve the ability to capture key regions and channels, we added a channel attention mechanism to our attention gate. We calculate the attention coefficient using:

Spatial attention:


(6) $$\begin{array}{l} {q^{l}}_{atts}=W_{2}\left(\sigma_{1}\left({W_{x}}^{T}x_{i}^{l}+{W_{g}}^{T}g_{i}\right)\right) \end{array}$$

(7) $$\begin{array}{l} \alpha_{i}^{l}=\sigma_{2}\left(q_{atts}^{l}\left(x_{i}^{l},g_{i};\Theta_{atts}\right)\right) \end{array}$$

where σ_1_(*x*_*i*_) = max(0, *x*_*i*_) represents ReLu function, $\sigma_{2}\left(x_{i},c\right)=\frac{1}{1+\exp\left(-x_{i},c\right)}$ denotes sigmoid function, and AG is represented by a set of parameters Θ_*atts*_, including the linear transformations $W_{x}\in {\mathbb{R}}^{F_{l}\times F_{int}}$, $W_{g}\in {\mathbb{R}}^{F_{g}\times F_{int}}$, and $W_{2}\in {\mathbb{R}}^{F_{int}}$. The linear transformations are realized by 1 × 1 convolution.

Channel attention:


(8) $$\begin{array}{l} {q^{l}}_{attc}=W_{1}\left(W_{0}\left(\sigma_{1}\left(AvgPool\left(x_{i}^{l}\right)+AvgPool\left(g_{i}\right)\right)\right)\right) \end{array}$$

(9) $$\begin{array}{l} \beta_{i}^{l}=\sigma_{2}\left(q_{attc}^{l}\left(x_{i}^{l},g_{i};\Theta_{attc}\right)\right) \end{array}$$

Different from spatial attention, in order to obtain the weight of each channel of the input feature map, the channel attention mechanism uses adaptive average pooling (the AvgPool part). In fact, adaptive average pooling works better than adaptive max pooling or use both of them in channel attention. The remaining linear transformations include two 1 × 1 convolutions: $W_{0}\in {\mathbb{R}}^{F_{g}\times F_{g/16}}$ and $W_{1}\in {\mathbb{R}}^{F_{g/16}\times F_{g}}$.

The output of the attention gate is:


(10) $$\begin{array}{l} x^{l}={\left\{\alpha_{i}^{l}\beta_{i}^{l}x_{i}^{l}\right\}}_{i=1}^{n} \end{array}$$

**Figure 6:**
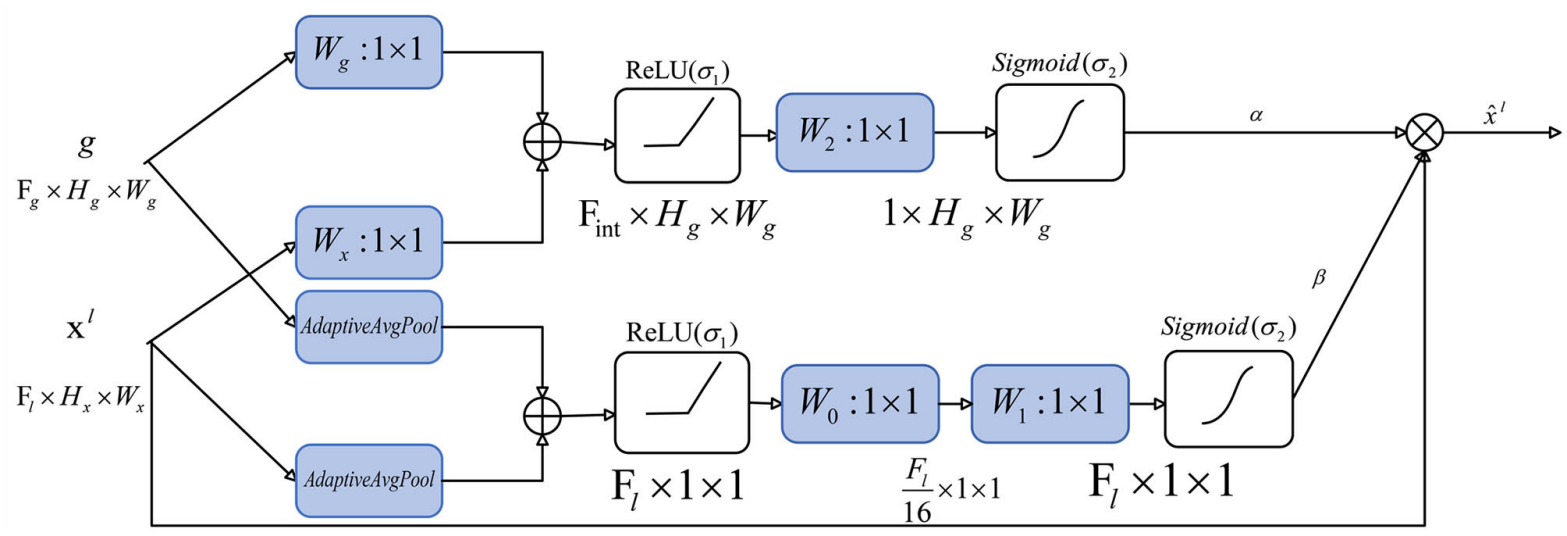
Attention gate.

The overall structure of our attention gate is shown in Figure 6:

The algorithm of the attention gate is as follows:

**Algorithm 2:**
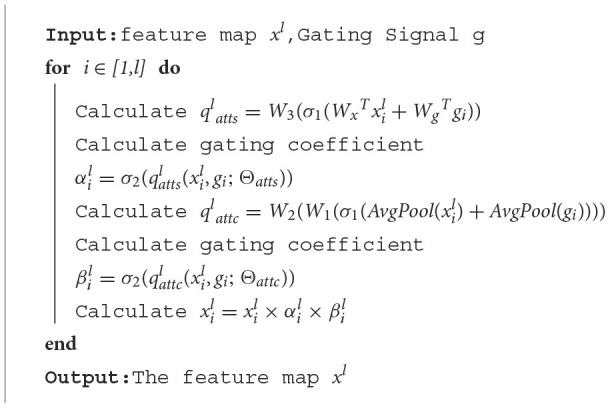
Attention gate.

## 6. Experiment

### 6.1. Metrics

For multi-class segmentation tasks, we used dice coefficients for each class as the main metric to measure the segmentation effect. Dice is the most frequently used metric in medical image competition. It is a set similarity metric, which is usually used to calculate the similarity of two samples, and the threshold is [0, 1]. It is often used for image segmentation in medical images. The best result of segmentation is 1, and the worst result is 0. The dice coefficient is calculated as follows:


(11) $$\begin{array}{l} Dice=\frac{2^{*}\left(pred\cap true\right)}{pred\cup true} \end{array}$$

where *pred* is the set of predicted values, *true* is the set of true values, the molecule is the intersection between *pred* and *true*, and the denominator is the union of *pred* and *true*.

In addition to the dice coefficient, we also applied PPV and sensitivity metrics to our experiment to further measure the segmentation result.

The definitions of PPV and sensitivity are as follows:


(12) $$\begin{array}{l} PPV=\frac{pred\cap true}{pred} \end{array}$$

(13) $$\begin{array}{l} Sensitivity=\frac{pred\cap true}{true} \end{array}$$

### 6.2. Experiment result

The experiment used the Task05 prostate data set in the medical decathlon competition. Due to the small number of experimental samples, we used offline data augmentation to enrich the training set. Specifically, we first performed data augmentation on prostate MRI images and corresponding labels, including horizontal/vertical flipping, rotation, adding Gaussian noise, and adjusting brightness and contrast, so that the amount of data in the training set is expanded to three times of the original set, which effectively alleviates the problem of small dataset and insufficient training data. Then, some operations are used for data preprocessing, including uniform image size, normalization, and slicing.

Among them, 80% of the original dataset and the pseudo image obtained by data augmentation are used as the training set, and 20 % of the original dataset is used as the validation set. In order to compare the performance of different networks, we trained the network proposed in this study and five other UNet based networks on the same dataset and under same hyperparameters as a comparison. All experiments are based on python language and pytorch framework, carried out on a server equipped with RTX308 and Windows11 system. The parameters of our experiment are shown in Table 1.

**Table 1:** Parameters of the experiment.

Loss	BCE dice loss
Epochs	1,000
Early stop	20
Batch size	4
Optimizer	Adam
Learning rate	0.0003
Momentum	0.9
Weight decay	0.0001

The experiment results of all networks are shown in Tables 2–4.

**Table 2:** Dice coefficient on the MSD prostate dataset.

**Network**	**Dice**	
	**PZ**	**TZ**
Unet	0.7061	0.863
Attention Unet	0.7785	0.8813
Res Unet	0.6623	0.8481
UNet++	0.6898	0.8837
R2AttentionUNet	0.4805	0.752
Proposed	**0.8070**	**0.9070**

The bold values indicate the best results across different networks.

**Table 3:** PPV on the MSD prostate dataset.

**Network**	**PPV**	
	**PZ**	**TZ**
Unet	0.7884	**0.9071**
Attention Unet	0.8648	0.8976
Res Unet	0.7971	0.8530
Unet++	0.7607	0.9043
R2AttentionUnet	0.6216	0.8282
Proposed	**0.8784**	0.9058

The bold values indicate the best results across different networks.

**Table 4:** Sensitivity on the MSD prostate dataset.

**Network**	**Sensitivity**	
	**PZ**	**TZ**
Unet	0.7887	0.8777
Attention Unet	0.7923	0.9074
Res Unet	0.7287	0.8992
Unet++	0.7831	0.9018
R2AttentionUnet	0.6191	0.7619
Proposed	**0.8254**	**0.9219**

The bold values indicate the best results across different networks.

From the tables mentioned above, it can be seen that the network we proposed in this study has achieved 0.807 and 0.907 dice scores in peripheral zone and transition zone of the prostate, respectively. It also achieved the best PPV and sensitivity scores. Compared with the other five UNet-based networks, the proposed method is better in the prostate segmentation task.

**Figure 7:**
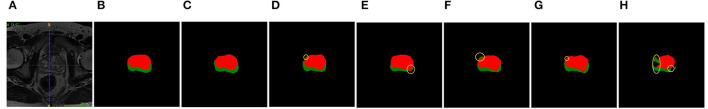
Segmentation maps of different networks. Left to right: **(A)** MRI image. **(B)** Ground truth. **(C)** MAS-UNet. **(D)** UNet. **(E)** Res UNet. **(F)** Attention UNet. **(G)** UNet++. **(H)** R2-Attention UNet.

The segmentation map is shown in Figure 7, in which the green area represents the peripheral zone (PZ) and red area represents the transition zone (TZ).

## 7. Discussion

Before determining the final network structure, the author has conducted a large number of comparative experiments to verify the effect of various modules on the data set used in this study. The results show that adding cyclic convolution and residual connections to the network does not make sense. When determining the pooling method for channel attention, comparative experiments have also been carried out. The results show that adaptive average pooling is better than adaptive maximum pooling or using both of them.

This study proposes a new prostate segmentation network based on the Unet framework. The network uses GN, ASPP, and channel attention to improve attention Unet, and uses different channels to output different label segmentation results.

We used Unet, attention Unet, Res Unet, Unet++, and R2AttUnet as five U-shaped networks for comparative experiments, and used the dice coefficient as an indicator to compare the effect of the model. The results show that the proposed model achieves 0.807 and 0.907 scores in the peripheral region and the transition region, respectively, and its segmentation effect is better than other classical U-shaped networks. MAS-UNet provides a new method for automatic prostate segmentation with higher accuracy than others, which would help to relieve the burden on radiologists.

While improving the segmentation effect, the network proposed in this study still has some defects: compared with the original Unet and Attention-Unet, the network proposed in this study increases the amount of calculation due to the introduction of some new modules, which makes the number of parameters of the model increase, and also creates higher requirements for hardware performance. Therefore, how to make the model as lightweight as possible under the premise of ensuring the existing segmentation accuracy will be one of the possible improvement directions in the future.

## Data availability statement

Publicly available datasets were analyzed in this study. This data can be found here: Medical Segmentation Decathlon (medicaldecathlon.com).

## Author contributions

YH performed the experiments and wrote the manuscript. ZQ offered guidance and corrected the writing of the manuscript. HC performed approval of the final version. BZ assistant in medical area and performed literature research. HL assistant in the experiment. All authors contributed to the article and approved the submitted version.
